# Exploring the prevalence and experience of mask anxiety for the person with head and neck cancer undergoing radiotherapy

**DOI:** 10.1002/jmrs.308

**Published:** 2018-10-30

**Authors:** Jodie L. Nixon, Bena Cartmill, Jane Turner, Amanda E. Pigott, Elizabeth Brown, Laurelie R. Wall, Elizabeth C. Ward, Sandro V. Porceddu

**Affiliations:** ^1^ Occupational Therapy Department Princess Alexandra Hospital Brisbane Australia; ^2^ School of Health and Rehabilitation Sciences University of Queensland Brisbane Australia; ^3^ Speech Pathology Department Princess Alexandra Hospital Brisbane Australia; ^4^ Centre for Functioning and Health Research Princess Alexandra Hospital Brisbane Australia; ^5^ Faculty of Medicine University of Queensland Brisbane Australia; ^6^ Radiation Oncology Princess Alexandra Hospital Ipswich Road Woolloongabba Australia

**Keywords:** Head and neck cancer, mask anxiety, radiotherapy, shell, thermoplastic mask

## Abstract

**Introduction:**

While use of a thermoplastic mask during radiotherapy (RT) treatment for head and neck cancer (HNC) is an essential component of safe patient care, there is little understanding of the extent to which this evokes anxiety (i.e. “mask anxiety”) for the person undergoing treatment.

**Methods:**

A mixed method, convergent design was used to examine the prevalence and experience of mask anxiety using two clinical cohorts. In phase one, a cohort of 100 patients undergoing RT for HNC were assessed for self‐perceived mask anxiety using a modified distress thermometer screening tool. In phase two, a separate cohort of 20 patients who identified as having mask anxiety participated in individual interpretative descriptive interviews to explore the nature of their experience.

**Results:**

In phase one, 26% of participants self‐identified as being anxious about the use of a thermoplastic mask. In phase two thematic analysis of the interviews revealed two over‐arching themes relating to the person's experience of mask anxiety: contributors to the mask anxiety (vulnerability, response to experience and expectations); and how the person was going to manage the mask anxiety during treatment (strategies and mindset).

**Conclusions:**

Mask anxiety impacted a quarter of participants undergoing radiotherapy for HNC. In line with the themes elicited from the participants, implementation of routine screening to ensure early identification, and patient education to assist preparation for wearing the mask during RT are strategies that could improve current management of mask anxiety.

## Introduction

To ensure precise radiotherapy (RT) delivery to sites of disease and avoidance of surrounding structures, patients with head and neck cancer (HNC) are immobilised during their treatment with a thermoplastic mask.^1^ Patients may experience anxiety and distress, which can impact on the accuracy, precision, and delivery of RT for HNC.^2^ There is little data about the prevalence of anxiety and distress associated with wearing a thermoplastic mask nor about the experience of the person who identifies as having mask anxiety during RT.

Use of the mask is unique to RT for HNC, brain and some upper chest cancers, and there are significant consequences if treatment cannot be delivered due to problems wearing the mask. In a previous study, patients with HNC identified wearing the mask was one of the worst parts of RT.^3^ In the only study conducted to date to explore rates of mask anxiety, the incidence of mask anxiety ranged from 16% to 24% (*n* = 70) depending on whether it was identified using a general distress tool (16%), or identification by a radiation therapist (24%).^4^ In research conducted by the same group, radiation therapists rated disruption to sessions on a scale of 1–5, and found an 11% rate of disruption to CT simulation and a 24% rate of disruption to Day 1 of treatment was attributed to mask anxiety.^2^

There has been limited exploration of the experience of mask anxiety for the person with HNC. One study interviewed a heterogenous cohort of 19 people immobilised for RT (only five participants were being treated for HNC) and found low anxiety associated with the mask, however, those with a pre‐existing phobia were more likely to experience distress.^5^ A pilot qualitative study of three HNC patients undergoing RT, revealed themes of physical discomfort of the mask; the mental perception of the mask; and, the passivity of accepting that it was part of the treatment, however, participants with mask anxiety were specifically excluded in that study.^6^ Thus, the paucity of evidence surrounding incidence of mask anxiety specific to HNC and the patient experience indicates the need for further exploration.

This study firstly aimed to examine the prevalence of mask anxiety for people undergoing RT for HNC and secondly explored the experience of the person who identifies as having mask anxiety.

## Methods

### Study design

This study used a mixed method convergent design which utilises both a quantitative and qualitative approach to explore mask anxiety.^7^ Phase one used a cross sectional quantitative design to determine the incidence of mask anxiety. Phase two employed a qualitative process of interpretative descriptive design to explore patient perceptions and experiences of mask anxiety to further understand the results of the phase one study.^8^ The two phases utilised independent recruitment processes and cohorts. This study was conducted at a single, quaternary hospital in Brisbane, Australia. Ethical clearance was received from the Metro South Hospital Research Ethics Committee (HREC/13/QPAH/437). All participants provided informed written consent.

### Participants

#### Phase One

Recruitment targeted a cross‐sectional sample of HNC patients who were booked for routine, weekly appointments with the multidisciplinary team (radiation oncologist, speech pathologist and dietitian). These participants were involved in a concurrent study investigating general distress, swallow and nutrition, and were scheduled to receive RT with curative‐intent treatment.^9^ Three one‐week periods of recruitment were conducted, with at least 6 weeks in between recruitment windows to ensure recruitment washout, thus limiting participants’ entering data more than once during the course of their RT. Patients with severe cognitive deficits, inability to read/write English, significant vision, hearing or physical dexterity impairments which would limit use of a self‐administered electronic screening tool were excluded from the study. Recruitment was conducted from October 2014 to May 2015.

#### Phase two

Recruitment occurred over a fixed 12‐month time frame between March 2016 and March 2017. Patients were included if they were having RT for HNC and identified distress/anxiety with the use of the mask either via the departmental, self‐administered electronic screening tool with a score greater than 4 of 10,^10^ or referral from nursing, medical or RT staff and were referred for occupational therapy intervention, as is standard care for mask anxiety in our centre. During the data collection period of phase two, our centre was implementing self‐administered electronic screening for HNC patients weekly during RT. As such, both standard care referrals from the MDT and electronic screening tool referrals were used as entry points into services for mask anxiety. During their initial occupational therapy appointment patients were offered the opportunity to participate in this study. Recruitment closed at the completion of the 12‐month period. Patients who had severe cognitive deficits or significant hearing impairments which would limit participation in an interview were excluded from the study.

### Measures

#### Phase one

**Figure 1:**
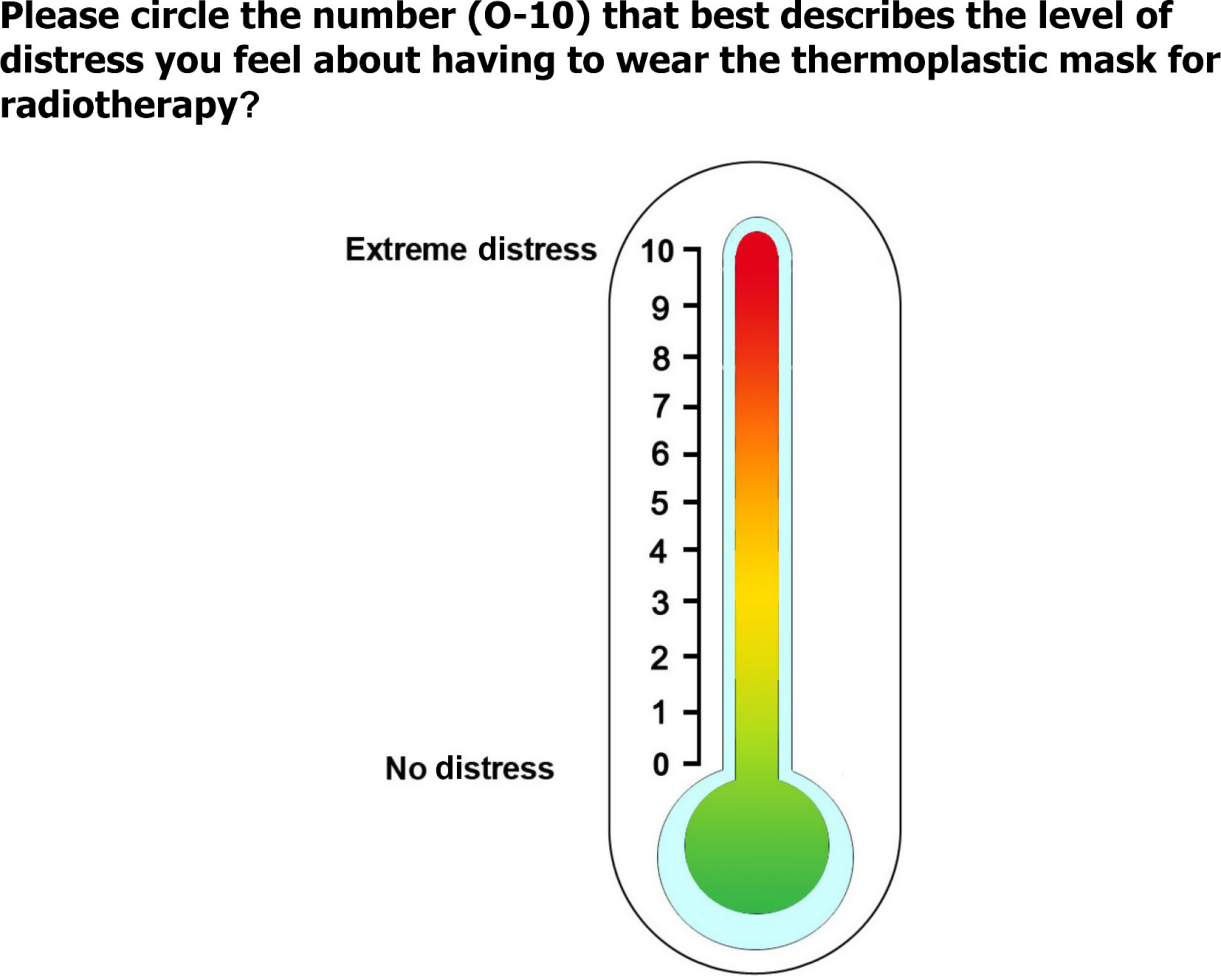
Mask distress thermometer.

No validated tool exists to measure mask anxiety. The distress thermometer (DT) is a widely used tool which has been validated for use in multiple cancer conditions and countries.^10^, ^11^ The DT was modified specifically to question distress related to mask anxiety: “Please circle the number (0–10) that best describes the level of distress you feel about having to wear the thermoplastic mask for radiotherapy?” (Fig. 1). As is consistent with the DT, a cut‐off score of 4 or above was considered a trigger for referral for support.^10^, ^12^ The DT modified to assess mask anxiety was completed at one time‐point between planning and completion of RT using a self‐administered electronic screening tool.

#### Phase two

A single semi‐structured interview was conducted either post‐planning, or during the first week of RT. This interview included open‐ended questions to explore the participant's experience of distress/anxiety related to the experience of being fitted for the mask at planning, and the preparedness to commence treatment.^13^ A two‐question interview guide, with exploratory probes was designed based on current literature and expert clinical knowledge (Appendix I). The questions were trialled on five patients and were considered to elicit information that was relevant to the research question. Interviews were conducted either by the principal investigator (JN) or a senior occupational therapist with both having extensive clinical experience with this cohort. The interviewer was not previously known to the participants. Interviews were conducted as per patient preference – via phone, face‐to‐face or with a carer present. All interviews were audio‐recorded and transcribed verbatim for analysis.

### Data analysis

#### Phase one

Descriptive statistics were used to analyse the participant characteristics and prevalence of mask anxiety. In the absence of any prior use of a validated tool to measure mask anxiety, or a mask specific DT, mask anxiety was considered if a score was equal or greater than 4 as is usual with the DT.^10^, ^11^, ^12^

#### Phase two

Interviews were transcribed verbatim by an independent research assistant. Thematic analysis was conducted by the research team – the principal investigator (JN) and another HNC specialist (BC) who was not involved in the interview process. Thematic checking was conducted by two other members of the research team (JT, AP) for consistency and consensus. Thematic analysis of the interviews was conducted using an interpretative descriptive design as proposed by Thorne.^8^ All team members have extensive experience working with the HNC population, the RT process, and the support required to manage mask anxiety, and this expertise was utilised as recommended by Thorne.^8^ Initial thematic analysis took place by concurrently listening to and reading the transcripts. A four‐step cognitive process of data analysis was undertaken: (1) comprehending the themes discussed by participants; (2) finding patterns that were similar; (3) reflecting and manoeuvring these findings for (a) the individual and (b) in relation to the group; and finally (4) reconceptualising how these themes may be used in other contexts and settings, and how to move this from theoretical contexts to that of clinical practice.^8^, ^14^ Initial themes were discussed in the research team, refined, and collapsed/expanded until consensus was reached. Simple percentages were used to reflect the proportion of the cohort who raised particular themes/subthemes.

## Results

### Phase one

Concurrent study participant details have previously been described in Wall et al.^9^ and their details were summarised in Table 1. One hundred participants were recruited over an 8 months time‐frame. A high percentage of participants were male (*n* = 80), with a mean age of 63 (SD = 12, range 34–93 years), most being treated for oral cavity/oropharyngeal cancers using surgery and post‐operative radiotherapy or concurrent chemoradiotherapy. All participants required the use of a thermoplastic mask including shoulder immobilisation during RT.

**Table 1:** Demographics of phase one and phase two

Demographic variable	Phase one	Phase two
No. participants	(*n* = 100) (%)	(*n* = 20) (%)
Age		
Mean	63 years	62 years
Range	34–93 years	30–83 years
Gender		
Male	80 (80)	15 (75)
Female	20 (20)	5 (25)
Surgery		
Yes	54 (54)	12 (60)
No	46 (46)	8 (40)
Radiotherapy		
PORT	54 (54)	12 (60)
CXRT	26 (26)	7 (35)
RT	20 (20)	1 (5)
Dose (Gy)	61.5 (range 30–70)	62.2 (range 55–70)
Number of fractions	30 (range 20–35)	30 (range 20–35)
Chemotherapy/systemic agent		
Cisplatin	18 (18)	5 (25)
Cetuximab	8 (8)	2 (10)
Referral source for mask anxiety		
Electronic screening tool	100	6 (30)
MDT		14 (70)
Interview mechanism	n/a	
Phone		19 (95)
Face to face		1 (5)
Interview source		
Participant		18 (90)
Participant carer		2 (10)
Duration interview (min)	n/a	Ave 17.45 (range 5–60)

PORT, post‐operative radiotherapy; CXRT, chemoradiotherapy; RT, radiotherapy; MDT, multidisciplinary team.

Cross‐sectional sampling for mask anxiety was completed through the course of RT, with participants having completed a mean of 64% of prescribed RT when recruited (SD = 19).^9^ The prevalence of patient‐reported anxiety associated with the use of the thermoplastic mask at any time during RT for HNC was 26%. Of this, 20% of participants indicated moderate distress (score 4–7 on the mask anxiety DT) and, 6% with severe distress (score 8–10 on mask anxiety DT).

### Phase two

**Figure 2:**
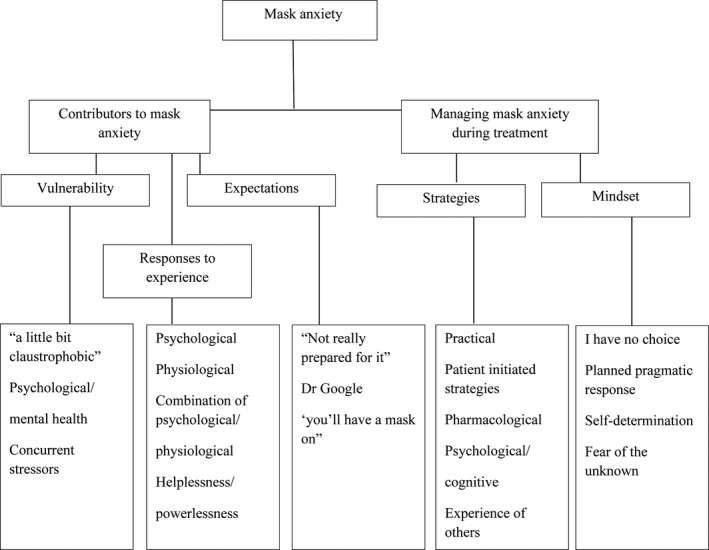
Overarching themes and subthemes.

A time‐convenience sample of 20 participants who were mostly male (*n* = 15), receiving post‐operative RT for HNC with mean age of 62 participated in an interview (Table 1). The majority of interviews (*n* = 19) were conducted over the phone and ranged between 5 min and 1 h in duration. Two participants had a carer present for the interview. Thematic analysis of the interviews revealed two over‐arching themes with five subthemes (Fig. 2). There were three subthemes that discussed the participants experience and contributors to the feelings of mask anxiety (Table 2), and two subthemes that discussed the participant's preparation for how they were going to manage the mask (Table 3).

**Table 2:** Themes related to contributors for mask anxiety

Theme	Subtheme	No. of participants (%)	Exemplar
Vulnerability	“a little bit claustrophobic”	14 (70)	P262: My main concern is that I suffer from claustrophobia P267: It was horrifying, I had no idea it would be like that and I'm claustrophobic at the best of times
	Psychological/mental health “a little bit panicky”	11 (55)	P275: Oh I just get, sorta, a little bit panicky, a little bit stressy P279: I have a history (depression). Yes, I do.
	Concurrent stressors	11 (55)	P267: Because now I am this person who is dealing with this bloody shitty thing and it just rules everything, it has taken over my life and let's face it, it has the potential to actually take my life which isn't much fun thinking about P269: So it's terminal so I've been in two minds whether to go along with it. It's in my spine now as well which isn't a good thing apparently. The ones in my head are under control but the one in my spine is going to get me.
Responses to experience	Psychological “it just freaked me out”	8 (40)	P256: And it'll probably freak me out again P277: But it's not too bad, you know, but I don't like it. I don't like it at all.
	Physiological “the sweating feelings”	6 (30)	P273: The sweating feelings, those sort of things and not having skilled myself up enough to focus on something else and just trying to get breathing back in my diaphragm and not in my chest where it was racing
	Combination of psychological/physiological “like I was having a heart attack”	12 (60)	P256: So the nerves are definitely going in the stomach, I've been in the toilet this morning P265: You don't see much, or you try to get your breath and you…and you start to panic
	Helplessness/powerlessness/no escape “it's like being buried alive”	10 (50)	P259: Being strapped down you know really got me worried P264: Just as long as they don't desert me there because I just get scared
Expectations	“Not really prepared for it”	6 (30)	P247 carer: No he wasn't really um prepared for that P255: It was probably more the… it was just unexpected
	Dr Google	3 (15)	P256: Doctor Google told me
	“you'll have a mask on”	2 (10)	P277: I just went in to see someone in charge of it and he said you'll have a mask on and, um, and that was virtually it.

**Table 3:** Themes related to managing mask anxiety during treatment

Theme	Subtheme	No. of participants (%)	Exemplar
Strategies	Practical “You just have to count”	14 (70)	P256: Susie Cotro (music)… she'll get me through it P264: They offered they can cut the eyes out for you
	Patient initiated strategies “I try….)	13 (65)	P269: I just sort of tried to control my breathing and I sort of just told myself to keep my breathing under control and reassure myself P272: I'm going fishing or when we get home we are going to the garden, to enjoy the garden or thinking about other things other than what is actually happening around me in the machine
	Pharmacological “The little tablet”	12 (60)	P259: I went back and saw my psychologist yesterday and he put me back on a tablet just to try and calm me down P255: I'd rather not take anything that I don't have to
	Psychological/cognitive “Once I'm more at ease”	10 (50)	P267: I'm a catholic so I have a prayer to St Jude that I say P273: I'm working on that with a lady using meditation who is taking me through ways of calming myself down
	Support of others “Just talking me the whole way through”	10 (50)	P267: Even just knowing that a human being was talking to you when you are in such a vulnerable position. P275: Plus, the staff in there were just awesome. They were laughing and joking and just talking me the whole way through it the whole time.
	Experience of others “A couple of friends have been through it”	3 (15)	P247 carer: She sort of um said to him that you know that it's awful and an awful thing to sort of think about, but when you actually get it done it's over and done with quickly, and it's not as bad as she'd thought it would've been herself at first
Mindset	Planned pragmatic response “Get my big boy pants on”	10 (50)	P255: Yes. No, I'll be fine on that first day. Yeah, I'll psyche myself up for it and be fine. P275: Just man up. It's something I've got to get over, I've got to get through, so yeah I've got to just do whatever it takes.
	I have no choice “Its got to be done”	3 (15)	P267: But I have no choice, that's the whole thing I don't have any choice
	Self‐determination “I'm determined”	3 (15)	P269: Look I pride myself on being pretty strong willed so I have to keep keeping on. Once I get my breathing under control and get myself settled I'll be able to get it done
	Fear of the unknown “Little bit the fear of the unknown”	2 (10)	P256: it is a little bit of the fear of the unknown, when you don't exactly know what to expect

### Themes related to the *participant experience* of mask anxiety

#### Theme 1

##### Vulnerability (Table 2)

Participants discussed three subthemes that contributed to their apprehension about managing the confinement of the mask for RT. The first was a pre‐existing claustrophobia (*n* = 14) that contributed to apprehension about how they would manage: “Yeah well it first started when I was doing some bricklaying by trade, and I did some bricklaying in the back of a boiler and because it is a confined space…. everything started closing in on me and I panicked and that is when I first noticed it” (P262). The second subtheme was that of pre‐existing psychological/mental health issues (*n* = 11) that contributed to anxiety/panic in new situations: “I guess my heightened awareness of the environment, is something I am aware of… I started off with knowing that my reaction is this situation was always going to be difficult to manage” (P273). The third subtheme participants discussed was concurrent stressors which either heightened their response to the mask or existed alongside the mask anxiety (Table 2). More than half of the participants (55%) discussed other stressors that were equally or more concerning than the mask. This ranged from concerns regarding side‐effects of treatment such as mouth opening and breathing, to practical issues such as housing and transport during treatment, and concerns regarding disease progression and whether the treatment would be effective.

#### Theme 2

##### Responses to experience (Table 2)

Participants discussed a combination of psychological and physiological responses to the process of having the mask made during the planning session: “When I'm…thinking about…the frame, head, mask thing (pause), the anxiety came up…and then the racing breath, you know, the sweating feelings” (P273). This response to the mask being made during the CT simulation was a contributor to the participants’ concerns about how they would manage the mask for the course of the RT. Participants also reported an emotional response of helplessness/powerlessness (Table 2). Half of the group reported feelings of not being in control, with one participant likened it to “being buried alive” (P279). Another described “I mean imagine someone behind you with their hand clamped across your mouth and you cannot open it or move it. It's an awful feeling” (P267). This description of not being in control of a situation was overwhelming for this group.

#### Theme 3

##### Expectations of treatment (Table 2)

Participants were aware they had to have the mask, but some still accessed “Dr Google” (*n* = 3) to find out more about the process. They felt unprepared regarding the experience of being fitted for the mask, or the process involved was unexpected: “They did tell me about it on a previous visit but I didn't realise it was so rigid and restrictive and that it is actually clipped to the bed of the scanner” (P267).

### Themes related to the *participant managing* mask anxiety during treatment

#### Theme 4

##### Strategies (Table 3)

There were six subthemes which related to strategies for managing the mask during the RT: (1) practical strategies – 70% of the group identified practical strategies that they were going to use, this included using music, having the eyes or mouth cut out of the mask, and waving their hand to stop treatment if needed; (2) patient initiated strategies (*n* = 13) – where participants discussed techniques that they were using/going to use, “I've been doing lots of self‐talk and you know, visualisation and I'm fine with it now”(P271); (3) pharmacological‐ 60% of participants discussed this as an option to help get through treatment, however, it is worth noting that 5 of 12 participants who discussed this did not want the medication as they did not like how it made them feel/could not drive/did not want to take anything extra; (4) psychological/cognitive strategies – participants discussed a number of ways that they were seeking external support to learn meditation/calming thinking, use of spiritual methods of prayer or using personal cognitive strategies; (5) support of others – 50% of participants discussed the value of support from others, this ranged from family members, doctor, but was mostly referencing the support of the radiation therapist talking to them during the planning/treatment process; and (6) experience of others – 15% of participants discussed that they had found it useful talking to other people who had been through the experience of using the mask.

#### Theme 5

##### Mindset (Table 3)

Participants discussed four different mindsets relating to their ability to manage the mask during RT. A larger number (50%), discussed a pragmatic response of self‐determination, “I understand that it's going to be up to me, I'm the only one that can put myself through it” (P259). Two participants talked about the experience of fearing the unknown with the use of the mask. Three others stated that they had no choice, “Yeah because what choice have I got? To just let it grow and strangle me? I can't do that” (P267).

## Discussion

Using the DT modified for mask anxiety, the study found that from a cohort of 100 people, 26% of patients identified distress with the mask, which is slightly higher than the previously reported prevalence.^2^, ^4^ This may be due to the use of a more targeted tool for data collection of mask anxiety, whereas previous studies have used more generalised anxiety measures, or it could be a reflection that mask anxiety may change through the treatment process, not isolated to CT simulation or Day 1 of RT as previously investigated.^2^, ^4^ In procedures where patients have felt confined, such as during MRI, it has been reported that 35–37% of people feel anxious.^15^, ^16^ With this level of anxiety and distress being experienced in cancer treatments and procedures requiring confinement, this is a phenomenon that needs closer examination of how to best support patients.

This study explored the experience of people undergoing treatment for HNC who identified as having mask anxiety, revealing two over‐arching themes: participants discussed how prepared they were for the mask fitting; and, they discussed the strategies they were going to implement to cope with the experience of having the mask during RT.

The first over‐arching theme relates to the contributors to mask anxiety. This group discussed their vulnerability of being claustrophobic, having pre‐existing psychological/mental health issues and other concurrent stressors as contributors to mask anxiety. These patient‐reported contributors are consistent with the five factors that Clover and colleagues reported which contributed to planning disruption in a group of 90 participants undergoing HNC treatment (taking psycho‐active medication, fear of enclosed spaces, fear of face covered up, fear of movement restriction and ever had an anxiety attack).^2^ As mask anxiety has been found in two sample populations, routine screening for mask anxiety may be warranted. Those affected may benefit from increased support either prior to planning or commencement of treatment. Future investigations are needed to examine whether proactive referrals better prepare patients for treatment resulting in less disruption during the RT process.

The second over‐arching theme is that of the strategies participants proposed to manage mask anxiety during the RT. The majority of participants discussed psychological strategies such as the use of self‐talk, relaxation and cognitive behavioural strategies and practical strategies such as eyes of the mask being cut out, and the use of music as being effective. Previous studies have supported the use of music as being beneficial to moderate anxiety level during radiotherapy.^17^, ^18^ Another strategy that participants reported as useful, with strong support in both RT and MRI literature is that of staff communication and providing concrete objective information about the process.^19^, ^20^ A study by Tazegul et al.^19^ in patients undergoing MRI showed that information and communication reduced anxiety both on a biochemical and psychiatric level. A clinical consideration would be to offer patients the opportunity to listen to music, access support or counselling, and for RT staff to be trained in communicating with anxious patients to help reduce and support the anxiety related to this procedure.

The use of medication is another strategy discussed by participants and recommended by other studies.^2^ However, a high proportion of participants discussed the preference to not use medication if not essential. A clinical consideration would be to use the above strategies either in conjunction with or as an alternate option to medication as per the person's request.

There are a number of limitations to this study. The prevalence of mask anxiety was established at different times during the RT process, which may not capture the extent of mask anxiety at all stages of treatment. Future studies should utilise a prospective data capture design at planning, week one, and then weekly during the course of treatment to observe patterns and trends. It is also recognised that mask anxiety prevalence in the current study has been determined using a modified mask anxiety DT measure in the absence of an existing validated tool. The interview process was offered at participant convenience, both in timing (i.e. pre‐treatment or during week 1) and delivery (i.e. face‐to‐face or phone, and with a carer present if requested by the participant), reducing the consistency of obtaining information. This variability in interview method allowed all participants access to accrual, and as such supported participant's involvement. The duration of interviews greatly varied, however, the interpretative descriptive model encourages all information to be accounted and included, regardless of content, so for this reason, all interviews were included, which supported the theme that a number of participants had concurrent stressors, which also need to be met to effectively support the person with HNC.

With close to one‐third of all people with HNC potentially having a negative experience of mask anxiety, screening for, and providing assessment/intervention should be considered as a standard care in this population. The current study serves to increase the awareness of specialists in the delivery of RT that mask anxiety is common and has broad‐ranging emotional and practical impacts. Further studies should seek to investigate the longitudinal patterns of mask anxiety during the course of RT treatment and explore patient perceptions of effective intervention.

## Conclusions

This study found that up to 26% of patients potentially experience mask anxiety. Treatment strategies that were proposed by participants to manage the mask anxiety consisted of a combination of psychosocial support, education from the treating staff and reassurance to the patient that the experience of mask anxiety is a normal response to being immobilised. These interventions require further research to determine their effectiveness, in supporting this group of people who experience mask anxiety while undergoing treatment for HNC.

## Conflict of Interest

The authors declare no conflict of interest.

## Appendix I Semi‐structured interview questions

**Table:**

1. Tell me about your experience in having the mask fitted and made during your planning session
*Exploratory questions:* Were you aware of the procedure required to fit and make the mask? Did you feel well‐prepared? How did the process of fitting and making the mask make you feel? Was there anything done during the process of fitting and making the mask that made you feel more at ease? More anxious/distressed? Have you got a history of claustrophobia? If yes, explore the history and onset of that How have you coped with situations in the past when you have been uncomfortable? Do you use any self‐initiated strategies to help you cope with the mask fitting and making? Did the staff suggest any strategies to help you cope with the mask fitting and making?
2**.** How do you feel about coming in for radiotherapy treatment and having to wear the mask daily to receive your treatment?
*Exploratory questions:* Do you feel well‐prepared? How do you feel you will cope? Are there any strategies or techniques you plan to use to make you feel more at ease?
